# Network Analysis of Transcriptome and LC-MS Reveals a Possible Biosynthesis Pathway of Anthocyanins in *Dendrobium officinale*

**DOI:** 10.1155/2020/6512895

**Published:** 2020-04-29

**Authors:** Zhiyao Ren, Fangning Qiu, Yinjie Wang, Wenxia Yu, Chenxing Liu, Yangyang Sun, Yawen Wang, Xiaofeng Zhang, Shangping Xing, Shengchang Tao, Yuechun Huang, Guoxiong Liu, Zhaofeng Wei, Baiyin Yu, Shuxiu Du, Zhouxi Lei, Gang Wei

**Affiliations:** ^1^School of Pharmaceutical Sciences, Guangzhou University of Chinese Medicine, Guangzhou 510000, China; ^2^The First Affiliated Hospital, Guangzhou University of Chinese Medicine, Guangzhou 510000, China; ^3^Shaoguan Institute of Danxia Dendrobium Officinale, Shaoguan 512005, China; ^4^Shaoguan Hejiantang Ecological Agriculture Co. Ltd., Shaoguan 512000, China; ^5^Shaoguan Danxia Mountain Engineering Center of Dendrobium Technology, Shaoguan 512000, China; ^6^Yingdong College of Life Science, Shaoguan University, Shaoguan 512005, China; ^7^Shaoguan Runhu Ecological Agriculture Co. Ltd., Shaoguan 512000, China; ^8^Guangzhou Baiyunshan Chenliji Pharmaceutical Co., Ltd., Guangzhou 510000, China

## Abstract

Anthocyanins, a group of flavonoids, are widely present in plants and determine the colors of the peels of stems, fruits, and flowers. In this study, we used UHPLC-ESI-MS to identify anthocyanins in the herbal plant *Dendrobium officinale*, which has been used for centuries in China. The results indicated that the total anthocyanin content in samples from Guangxi was the highest. Seven anthocyanins were identified, and the fragmentation pathways were proposed from *D. officinale*. Most of the identified anthocyanins were composed of cyanidin and sinapoyl groups. We also carried out that the sinapoyl group had active sites on breast cancer receptors by using Schrödinger. The relative levels of the 7 anthocyanins in the samples from the three locations were determined. Transcriptomic analysis was used to analyze the sinapoyl anthocyanin synthesis-related genes in plants, such as genes encoding UGTs and serine carboxypeptidase. We speculated that sinapoyl anthocyanin biosynthesis was associated with the activities of certain enzymes, including chalcone flavonone isomerase-like, hydroxycinnamoyltransferase 1, UGT-83A1, UGT-88B1 isoform X1, serine carboxypeptidase-like 18 isoform X3, and serine carboxypeptidase-like 18.

## 1. Introduction

*Dendrobium officinale* is a widely used herb in China and southern Asian countries. This plant was first recorded as a traditional Chinese medicine in *Shennong's Classic of Materia Medica*, which was written more than 2000 years ago [1, 2]. The stems of *D*. *officinale* provided the benefits of improving body immunity, promoting salivation, antineoplasticity, and regulating blood sugar level because of large amounts of polysaccharides, flavonoids, alkaloids, and bibenzyl compounds [3–5]. With the increasing demand for human beings, this plant became nearly extinct in the wild and is now a second-class protected species in China. Currently, most *D. officinale* in the pharmaceutical markets is obtained by artificial cultivation in South China [6].

Anthocyanin, a type of flavonoids, is one of the most important secondary metabolites in plants. The concentration and type of those compounds determine the colors of plants [7]. Most reports on anthocyanins have focused on different kinds of flowers and the peels or seeds of fruits, such as grape [8]. Modern studies have shown that anthocyanin exhibits many types of bioactivity in vitro and in vivo, for example, antimicrobial [9], antidiabetic [10], anti-inflammatory [11], antioxidant [12], and anticancer [13, 14]. Anthocyanin use is gaining popularity, and many food companies have developed several types of anthocyanin products for health preservation, such as grape seed extract, which is an important raw material in the food industry [15]. However, there remains a lack of studies on anthocyanins in other important plants. A preliminary study by our research group revealed that there exist significant variations in *D. officinale* based on place of production, such as the quantity and quality of flavonoids [16]. Notably, the appearance of the plant, especially the color of the peel, can differ. A previous report has stated that there exist substantial differences in anthocyanin content between *D. officinale* plants with green and red peels. Peel color is determined by the anthocyanin present in the stem of this plant [17]. But the related reports of qualitative differences of *D. officinale* among different areas are rare. Those genes involved in regulating the red peels are still defective.

Therefore, in this study, we used the UHPLC-MS/MS system to compare the quantitative and qualitative differences of *D. officinale* among the Guangdong, Guangxi, and Zhejiang provinces. For the identified anthocyanins, we aimed to propose the compound-related disease gene and analyze the active site of the disease-related gene at the molecular level by molecular docking. Base on a certain pharmacological effect of the identified anthocyanin, it is necessary to understand the biosynthesis of anthocyanin in plants. Thus, the biosynthesis-related genes in anthocyanin pathways of *D. officinale*, including UGTs and serine carboxypeptidase, were analyzed by transcriptome.

## 2. Materials and Methods

### 2.1. Reagents

The reagents used in this study were acetonitrile (Merck, Germany, chromatography grade, lot number: 20170703312), formic acid (Tianjin Zhiyuan Chemical Products Ltd., lot number: 20170721314), hydrochloric acid (Tianjin Zhiyuan Chemical Products Ltd., lot number: 20171008278), ethanol (Tianjin Zhiyuan Chemical Products Ltd., lot number: 2016110132), and distilled water (self-prepared).

### 2.2. Plant Materials

**Figure 1:**
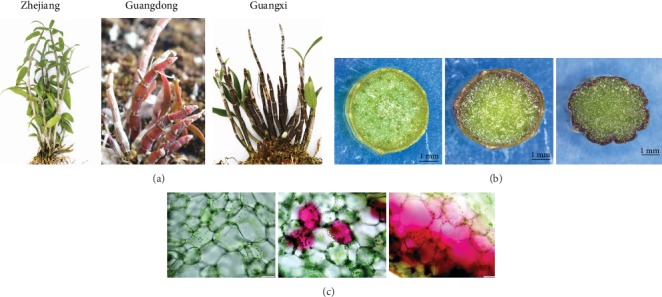
(a) The graph of the appearance of the *D. officinale* from 3 different places; (b) the transverse section of the stems; (c) the micrograph of the pigments in the epidermal cells of the stem.

Nine batches of fresh, mature samples of the plant *D. officinale* were collected from 3 different places of production in Guangdong (GD), Zhejiang (ZJ), and Guangxi (GX) provinces (Figure 1). As shown in Figure 1, the peels of the samples from Guangxi province were more purple than those from Guangdong while the samples from Zhejiang province were green.

### 2.3. Extraction of *D. officinale* Anthocyanins

Ten grams of fresh *D. officinale* stem was weighed precisely and cut into pieces. 1000 mL of 90% ethanol with 1% hydrochloric acid was added to the samples in a conical flask. The extraction was performed in the dark for 24 h. The liquid from the conical flask was filtered to obtain anthocyanin extract from the residue. The extract was dried at 40°C using a rotary evaporator in the dark. Ethanol was added to dissolve the extract and bring it to a 5 mL volumetric flask. A 0.22 *μ*m filter membrane was used to obtain the final anthocyanin extract, which was used for quantitative and qualitative analyses.

### 2.4. Determination of the Total Anthocyanin Content in *D. officinale*

The total anthocyanin content in *D. officinale* was calculated by the pH differential method. The formula used for calculation was as follows:

(1) $$\begin{array}{c} \text{Anthocyanin content }\left(\text{mg}/\text{mL}\right)=\frac{A\times \text{MW}\times \text{DF}}{\varepsilon \times W}, \end{array}$$

where *A* is the absorption at a specific wavelength, calculated as follows:

(2) $$\begin{array}{c} A=\left(A520-A700\right)\text{pH}1.0-\left(A520-A700\right)\text{pH}4.5. \end{array}$$

(*A*520 − *A*700) pH 1.0 refers to the absorption of the sample solution at 520 nm minus the absorption at 700 nm when the pH of the sample solution is 1.0; (*A*520 − *A*700) pH 4.5 refers to the absorption of the sample solution at 520 nm minus the absorption at 700 nm when the pH of the sample solution is 4.5.

MW in the first formula refers to the molecular weight of cyanidin 3-glycoside, which was equal to 449.2; DF refers to the dilution factor of the sample solution; *ɛ* refers to the molar absorption coefficient, which was equal to 26900; and *W* refers to the weight of the sample.

### 2.5. Composition and Relative Quantitative Analysis by UHPLC-MS/MS

UHPLC-MS/MS analysis was performed on an HPLC with a UV detector (Thermo Separation Products Inc., Riviera Beach, FL, USA) system equipped with a Thermo Finnigan LCQ FLEET (Thermo Finnigan, Riviera Beach, FL, USA) ion trap mass spectrometer as well as an ESI source. The column used in this study was an Agilent Zorbax SB-Aq (250 mm × 4.6 mm, 5 *μ*m). The mobile phase was composed of A (acetonitrile) and B (1% formic acid water) using a gradient elution: 0~15 min, 15~17% A; 15~20 min, 17~18% A; 20~35 min, 18~20% A; and 35~40 min, 20~25% A. The wavelength for UV detection was 520 nm; the flow rate was set at 1.0 mL/min; the column temperature was 35°C; the injection volume was 4 *μ*L.

The settings for the ESI source was as follows: the analysis was performed in a positive mode ([M+H]^+^), capillary temperature at 320°C, sheath gas flow rate at 40 psi, Aux gas flow at 2.0 psi, capillary voltage at 4.2 kV, and tube lens at 90.00 kV. The mass range was from 50 to 1900 *m*/*z*.

The relative quantitative analyses of all the identified anthocyanins were performed by calculating the peak area ratios of the target and the largest peak at 520 nm, which could be used to distinguish anthocyanins from flavonoids and other phenolic compounds. The qualitative analyses of anthocyanins were performed by data-dependent MS^n^ scanning to trigger the target ion fragmentation and prevented repetition.

### 2.6. Computational Target Fishing

The targets of the identified anthocyanins were searched from the Swiss target prediction database (http://www.swisstargetprediction.ch/). The names of proteins and their IDs were obtained on UniProt (https://www.uniprot.org/) under the condition of “homo sapiens.” The targets of disease were collected from GeneCards Human Gene Database (https://www.genecards.org/) and the Online Mendelian Inheritance in Man (OMIM, https://omim.org/).

### 2.7. Molecular Docking

To evaluate the predicted targets, the candidate protein crystal structures were downloaded from the RCSB Protein Data Bank (https://pdb.org/). The sdf files of ligands were obtained from ChemDraw 18.0 software. Docking simulations were carried out by Maestro 11.9 (Schrödinger, LLC, Cambridge, USA).

### 2.8. Transcriptomic Analysis of Anthocyanidin Biosynthesis-Related Genes

In this study, we used the transcriptomic data published by our research group in 2018 to perform all transcriptomic analyses [16]. Due to anthocyanidins and flavonoids sharing the same biosynthesis pathway in the KEGG database, we aimed to compare the expression levels of anthocyanidin synthesis-related genes and flavonoid synthesis-related genes. In addition, we also compared genes encoding UDP-glycosyltransferase and serine carboxypeptidase, which are involved in anthocyanin synthesis and led to the component diversity. Gene expression levels are shown as FPKM values.

### 2.9. Correlation Analysis of Metabolites and Transcriptome Data

The Pearson correlation coefficient was used to perform the association analysis of transcriptomic and related anthocyanin (Pearson correlation coefficient > 0.8 as a significant correlation) [18, 19]. The relationships between metabolites and related genes were visualized by using Cytoscape 3.7.2 (The Cytoscape Consortium, San Diego, USA).

## 3. Results

### 3.1. Identification of Anthocyanins in *D. officinale*

**Figure 2:**
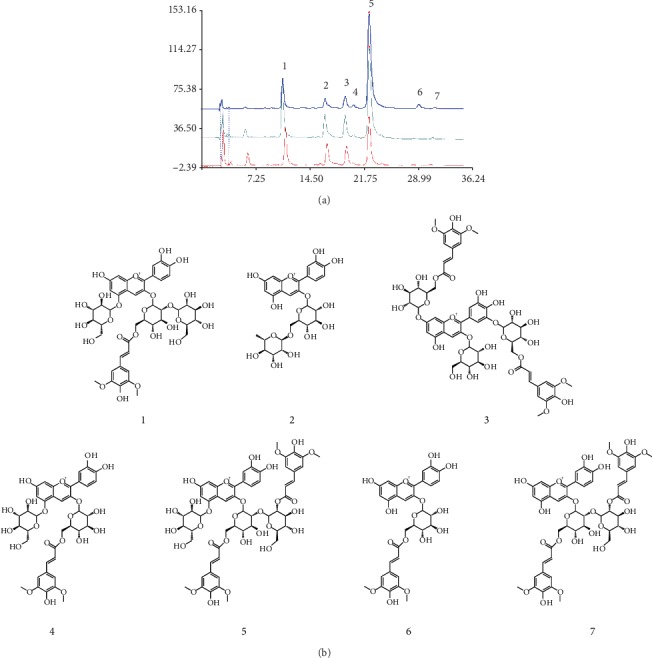
The anthocyanin composition in *D. officinale*. (a) HPLC of seven anthocyanins at 520 nm; (b) the chemical structures of the seven anthocyanins.

**Figure 3:**
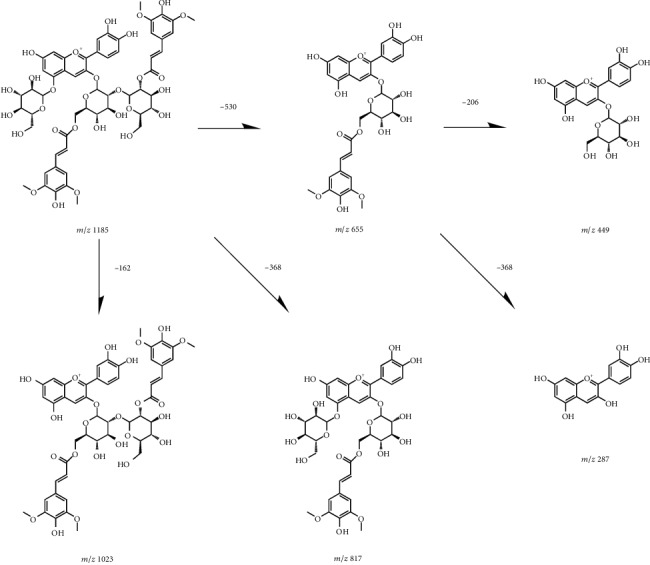
The proposed fragmentation pathways of peak 5.

The anthocyanin composition of *D. officinale* is shown in Figure 2. Twelve peaks were presented at a wavelength of 520 nm. Seven of these peaks were identified by the MS^2^ fragmentation. The fragmentation regular of each identified peak is shown in Table 1. The MS^2^ spectrum fragmentations of 7 anthocyanins are shown in Figures S1–S7. Six of the 7 identified anthocyanins consisted of cyanidin while one of them consisted of delphinidin. In addition, there was a sinapoyl group in nearly all the identified anthocyanins. The sinapoyl group was attached to a glycoside. The ESI-MS results showed that there were three kinds of prominent fragment ions, such as characteristic sugar-related product ions [M+H-162]^+^, sinapoylglucoside-related product ions [M+H-368]^+^, and sinapoyl-related product ions [M+H-206]^+^ in the positive ion mode. The proposed fragmentation pathways of peak 5 are shown in Figure 3. The identification processes based on regular fragmentation of the anthocyanins were described below.

Peak 1 was identified as cyanidin 3-[2-(glucosyl)-6-(sinapoyl)glucoside]-5-glucoside [20, 21], C_44_H_51_O_25_^+^, which exhibited a retention time of 10.944 min by UV chromatography. The molecular weight of this compound was 979.01, and the peak was fragmented to 817.12 and 655.05 by MS^2^; the ion peak at 655.05 was fragmented to 449.09 and 287.09. The peak at 287.16 was attributed to cyanidin, while the peak at 449.09 was attributed to cyanidin-5-glucoside. The peak at 817.02 was attributed to cyanidin 3-[6-(sinapoyl)glucoside]-5-glucoside.

Peak 2, at 16.604 min, in the UV chromatogram was identified as cyanidin 3-O-rutinoside [17], C_27_H_31_O_15_^+^. The molecular ion peak was at 595.05, which was fragmented to 433.05, 415.05, 397.13, and 295.05.

Peak 3, at 19.285 min, was identified as delphinidin 3-glucoside-7,3′-di-[6-(sinapoyl)glucoside] [22], C_55_H_61_O_30_^+^. The molecular ion peak was at 1202.04, and this peak was fragmented to 832.96. The fragment ion at 832.96 was fragmented to 671.07, 517.05, 465.04, and 303.18. The ion peak at 303.18 was identified as delphinidin, while the ion peak at 465.04 was attributed to delphinidin 3-O-glucoside.

Peak 4, at 20.380 min, was characterized as cyanidin 3-[6-(sinapoyl)glucoside]-5-glucoside [23], C_38_H_41_O_20_^+^. The molecular ion peak was at 817.05, as described above, and this peak at 817.05 was characterized as cyanidin 3-[6-(sinapoyl)glucoside]-5-glucoside. In addition, the regular fragmentation pattern was similar to that of the peak at 10.944, which showed an analogical structure.

Peak 5, at 22.483 min, was characterized as cyanidin 3-[6-sinapoyl-2-O-(2-(sinapoyl)glucosyl)-glucoside]-5-glucoside [24–26], C_55_H_61_O_29_^+^. The molecular ion peak was at 1185.01, and the peak was fragmented to 1022.94, 817.09, 654.91, 449.09, and 287.06. *m*/*z* 817.09 was deduced to lost sinapoylglucoside-related product ion from cyanidin 3-[6-sinapoyl-2-O-(2-(sinapoyl)glucosyl)-glucoside]-5-glucoside to cyanidin 3-[6-(sinapoyl)glucoside]-5-glucoside. *m*/*z* 654.91 was concluded to lost sugar-related product ion [M+H-162]^+^ as well as sinapoylglucoside-related product ion [M+H-368]^+^. Thus, the peak at 449.09 could be attributed to cyanidin-3-glucoside for the sinapoyl group lost from cyanidin 3-[6-(sinapoyl)glucoside] during fragmentation.

Peak 6, at 29.950 min, was characterized as cyanidin 3-[6-(sinapoyl)glucoside] [24], C_32_H_31_O_15_^+^, with a molecular ion peak at 655.05, which was fragmented to 449.03 and 287.11. As described above, the peak at 287.11 could be attributed to cyanidin. The sixth peak and the second peak were similar, differing in only the sinapoyl group at position 6 of the cyanidin 3-glycoside.

Peak 7, at 30.945 min, was characterized as cyanidin 3-[6-sinapoyl-2-O-(2-(sinapoyl)glucosyl)-glucoside] [24], C_49_H_51_O_24_^+^, with a molecular ion peak at 1022.8. The fragmentation pattern showed peaks with *m*/*z* values of 655.14 and 449.27. Obviously, the peak at 449.27 was attributed to cyanidin-3-glucoside.

### 3.2. Total and Relative Anthocyanin Content in *D. officinale*

**Figure 4:**
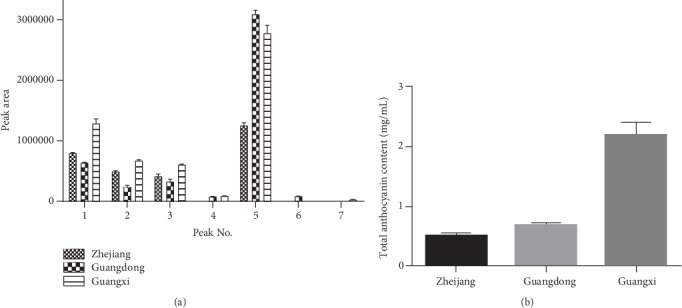
(a) The relative content of the identified anthocyanins; (b) the total anthocyanin content in samples from 3 different places.

The total anthocyanin content of *D. officinale* from Zhejiang, Guangdong, and Guangxi provinces is shown in Figure 4(b). The average total anthocyanin content in the plant from Guangxi, Guangdong, and Zhejiang provinces was 2.144, 0.648, and 0.532 mg/mL. The total anthocyanin content in *D. officinale* from Guangxi province was the highest. The relative content of anthocyanin determined by a peak area in *D. officinale* is shown in Figure 4(a). There were only 4 peaks observed for plants from Zhejiang province while there were 6 peaks for plants from Guangdong and Guangxi provinces, respectively. Based on the relative peak sizes, the relative anthocyanin content for different provinces exhibited diverse characteristics. The relative content of cyanidin 3-[6-sinapoyl-2-O-(2-(sinapoyl)glucosyl)-glucoside]-5-glucoside was much higher than the other six peaks in plants from each province. Cyanidin 3-[6-(sinapoyl)glucoside] was present in samples from Guangdong province, while cyanidin 3-[6-sinapoyl-2-O-(2-(sinapoyl)glucosyl)-glucoside] was present in samples from Guangxi province. Cyanidin 3-[6-(sinapoyl)glucoside]-5-glucoside was present in samples from Guangdong and Guangxi provinces but not in samples from Zhejiang province.

### 3.3. Analysis of Anthocyanin Target Gene

All the identified anthocyanin molecular formulas were sent to the Swiss target prediction database to get 105 targets. 67 targets were retained after removing duplicates and correcting the protein name under the condition of “homo sapiens” in the UniProt database. Combining GeneCards and OMIM databases, the top 500 of total 13268 targets were finally chosen as the breast cancer-related targets. Ultimately, 21 common targets of the seven identified anthocyanins and breast cancer were found. The common targets were TYMS, CA9, VEGFA, FGF1, CTSD, FGF2, MMP13, MMP1, TNF, TOP1, LGALS3, ABCC1, ABCB1, ABCG2, PTGS2, PRKCA, JUN, PARP1, HRAS, ERBB2, and EGFR. The results of 7 anthocyanins and their related targets are shown in Table S1.

### 3.4. Molecular Docking Analysis

**Figure 5:**
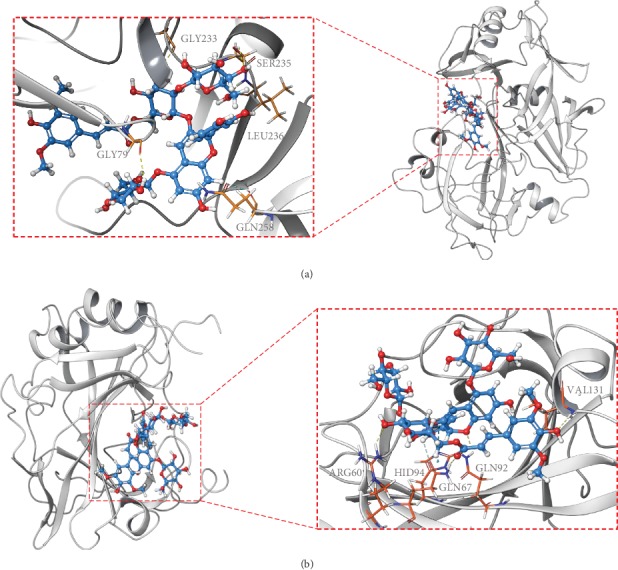
Schematic (3D) representation that the molecular model of the ligand combined with the targets. Ligand: cyanidin 3-[2-(glucosyl)-6-(sinapoyl)glucoside]-5-glucoside. Protein: (a) CTSD; (b) CA9.

The potential interactions of the active compounds and disease-related genes were clarified by molecular docking. CTSD and CA9 frequently appeared in identified anthocyanins with a count of four and three, respectively. Cyanidin 3-[2-(glucosyl)-6-(sinapoyl)glucoside]-5-glucoside was the second highest compound of the seven anthocyanins. Thus, they were chosen as the proteins and ligand in this docking. The results showed that cyanidin 3-[2-(glucosyl)-6-(sinapoyl)glucoside]-5-glucoside was successfully docked into the breast cancer-related proteins, CTSD and CA9. The dock details are shown in Table 2. The binding mode of cyanidin 3-[2-(glucosyl)-6-(sinapoyl)glucoside]-5-glucoside in the active site of CTSD (PDB:4OD9) and CA9 (PDB:6FE0) is represented in its three-dimensional mode in Figure 5. In the active site of CTSD, the glycosyl group showed H-bond interactions with GLY233 (3-glucoside), SER235, LEU236 (2-glucoside), and GLY79 (5-glucoside). A hydrogen in flavonoid skeleton A-ring showed aromatic H-bond with GLN285. In the active site of CA9, we surprisingly found that the carbonyl and hydroxyl in the sinapoyl group had H-bond interactions with GLN67 and VAL131. Flavonoid skeleton B-ring showed not only H-bond interactions but also pi-pi stacking with HID 94. Oxygen in flavonoid skeleton C-ring showed H-bond with GLN92 and in 3-glucoside interacted with ARG60.

### 3.5. Transcriptomic Analysis of the Anthocyanidin Synthesis-Related Pathway

**Figure 6:**
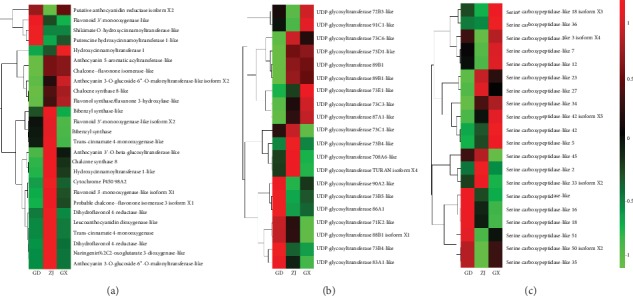
(a) The expression of genes related to anthocyanidin synthesis; (b) the heat map of UGTs; (c) the heat map of serine carboxypeptidase genes in *D. officinale*.

The expression levels of anthocyanidin synthesis-related genes and genes involved in the flavonoid synthesis pathway were evaluated. The heat map of the annotated genes is shown in Figure 6(a). A total of 26 genes were annotated as being in the flavonoid synthesis pathway based on the KEGG database, and 3 of these genes were closely related to anthocyanidin synthesis, encoding anthocyanidin 3′-O-beta-glucosyltransferase and anthocyanidin 3-O-glucoside-6^″^-O-malonyltransferase, anthocyanidin 5-aromatic acyltransferase. Anthocyanidin 3′-O-beta-glucosyltransferase-like is a specific protein that glucosylates the 3′-hydroxy group of delphinidin 3,5-di-O-glucoside to produce gentiodelphin. Anthocyanidin 3-O-glucoside-6^″^-O-malonyltransferase can transfer the malonyl group from malonyl-CoA to cyanidin 3-O-glucoside [27]. The enzyme anthocyanidin 5-aromatic acyltransferase is involved in the malonylation of the 5-O-glucose residue of anthocyanidins [28]. Besides, 3 genes were relative to anthocyanin content. Chalcone flavonone isomerase not only is a key enzyme in the flavonoid synthesis pathway but also can regulate the accumulation of anthocyanins [29]. Hydroxycinnamoyltransferase catalyzes the transfer of an acyl from p-coumaroyl-CoA to various acyl acceptors, such as anthocyanin O-hydroxycinnamoyltransferase [30, 31]. Anthocyanidin reductase is an important enzyme to convert cyanidin to (-)-epicatechin [32]. Based on the heat map, anthocyanidin 3-O-glucoside-6^″^-O-malonyltransferase-like isoform X2, anthocyanidin 5-aromatic acyltransferase, chalcone flavonone isomerase-like, and hydroxycinnamoyltransferase 1 were highly expressed in samples from Guangxi province, where the total anthocyanin content was high.

### 3.6. Expression Level of UGTs

According to the heat map, there were no regular trends in the expression of UDP-glycosyltransferase; the samples from three different locations exhibited different expression patterns. The heat map of UGTs is shown in Figure 6(b). For example, UGT-90A2, UGT-73B5, and UGT-86A1 were highly expressed in samples from Guangdong province, while UGT-73C1, UGT-73B4, and UGT-708A6 were highly expressed in samples from Zhejiang. UGT-73E1, UGT-73C3, and UGT-71A1 were highly expressed in samples from Guangxi. The main function of UGTs is to catalyze the addition of glycosyl groups to other molecules. Some UGTs identified in this study were flavonoid glycosyltransferases. For example, the main function of UGT-73B3 is to synthesize 3-O-flavone in vitro [33]. The protein UGT-708A6 was reported to catalyze the conversion of glucose to 2-hydroxynaringenin [34]. The expression of these UGTs was certainly associated with anthocyanin synthesis in *D. officinale*. Based on the FPKM values, some UGT expression levels were high such as UGT-91C1, UGT-83A1, and UGT-88B1 isoform X1. UGT-91C1 was highly expressed in samples from Guangxi province, while UGT-83A1 and UGT-88B1 isoform X1 were highly expressed in samples from Guangdong.

### 3.7. Expression Level of Serine Carboxypeptidases

Carboxypeptidases are a group of proteins with acyltransferase function. However, some studies have shown that certain types of serine carboxypeptidase can catalyze the formation of 1,2-bis-O-sina*p*oyl-*β*-D-glucoside, such as serine carboxypeptidase-like 8, serine carboxypeptidase-like 19, and serine carboxypeptidase-like 9 [35]. Although all serine carboxypeptidases have not been reported to be associated with the synthesis of sinapoyl in anthocyanins, we believe that certain highly expressed serine carboxypeptidases should be investigated. In this study, 20 genes annotated as encoding serine carboxypeptidases are shown in Figure 6(c). 14 genes were relatively highly expressed in samples from Guangxi, including serine carboxypeptidase-like 18 isoform X3, 36, and 3 isoform X4. Five genes, including serine carboxypeptidase-like 27, 2, and 33 isoform X2, were relatively highly expressed in samples from Zhejiang province, while six genes, including serine carboxypeptidase-like, 16 and 18, were highly expressed in samples from Guangdong.

### 3.8. Correlation Analysis of Metabolites and Related Genes

**Figure 7:**
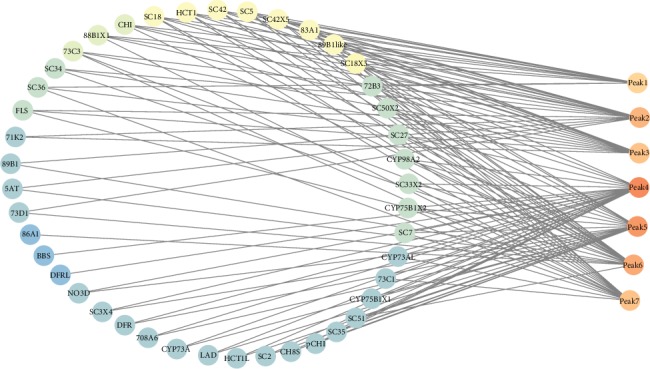
Connection network of metabolites and related genes. Low values of Pearson correlation coefficient to dark colors (blue); high values of Pearson correlation coefficient to bright colors (yellow).

The association analysis between 7 identified anthocyanins and transcripts was carried out. Eight transcripts were selected to be strongly correlated (Pearson correlation coefficient > 0.8, *p* value < 0.01) to the 7 metabolites. 19 transcripts were chosen as related genes (0.8 > Pearson correlation coefficient > 0.668, 0.01 < *p* value < 0.05) to the identified metabolites (Table S2). As shown in Figure 7, high values of the Pearson correlation coefficient to bright colors (yellow), some UGTs, and serine carboxypeptidases such as UGT-89B1-like, UGT-83A1, and UGT-88B1 isoform X1 serine carboxypeptidase 5, 18, 42, and 18 isoform X3 had a strong correlation with the content of peaks 1-7. Except for the UGTs and serine carboxypeptidases, the genes on the anthocyanin pathway such as chalcone flavonone isomerase-like and hydroxycinnamoyltransferase 1 also had a correlation to the anthocyanin components.

## 4. Discussion

Anthocyanins are the main determinants of the colors of the plants' tissue [36]. Normally, these compounds are present as glycosides. This is the first report that the structures of anthocyanins in *D. officinale* are present not only with glycosides but also with sinapoyl groups. Thus, the total anthocyanin content and the relative quantities of these identified anthocyanins were determined. Although the relative quantities of the samples from the three areas were different, the compositions of the anthocyanin have certain similarities. For example, cyanidin 3-[6-sinapoyl-2-O-(2-(sinapoyl)glucosyl)-glucoside]-5-glucoside was present at the highest level in all the samples.

To analyze the pharmacological activities of the identified anthocyanins, we found that all of them could act on breast cancer-related targets from the compound and disease database. Both CTSD and CA9 are the important targets that have reported the therapeutic effects on breast cancer [37–40]. At the same time, increasing studies have reported the therapeutic effect of anthocyanins on breast cancer, but they mainly focused on cyanidin-3-glucoside [41, 42]. In this study, we found that the sinapoyl of cyanidin 3-[2-(glucosyl)-6-(sinapoyl)glucoside]-5-glucoside provided active sites on the breast cancer-related protein by Schrödinger docking. It may be one of the pharmacologically active groups of anthocyanins.

Based on the pharmacological activities of anthocyanins, the synthetic pathway of anthocyanin in *D. officinale* needs to be clarified, which can provide the basis for the transformation of medicinal plants. Sinapoyl anthocyanins are widely present in many plants, such as *Arabidopsis thaliana* [43] and *Orychophragonus violaceus* [44]. However, the sinapoylation of anthocyanins in plants has not been studied extensively.

The biosynthesis of cyanidin and delphinidin was attributed to structural genes in the anthocyanin pathway. It can be speculated that the expression levels and correlation of the genes in the anthocyanin synthesis pathway determined the total anthocyanin content. We concluded that anthocyanidin 3-O-glucoside-6^″^-O-malonyltransferase-like isoform X2, anthocyanidin 5-aromatic acyltransferase, chalcone flavonone isomerase-like, and hydroxycinnamoyltransferase 1 were closely related genes, especially chalcone flavonone isomerase-like and hydroxycinnamoyltransferase 1. The high expression level of these genes in samples from Guangxi contributed to the higher total anthocyanin content in samples from Guangxi than others. Although anthocyanidin reductase, an important enzyme to convert cyanidin to (-)-epicatechin [32], was expressed low in samples from Zhejiang, it might lead to the low content of (-)-epicatechin. Due to the high total anthocyanin content in Guangxi, it was possible that anthocyanidin reductase could not dramatically transform cyanidin to (-)-epicatechin. Many structural genes were highly expressed in Zhejiang province based on the heat map, but a majority of them were in the flavonoid synthesis pathway without a significant correlation of anthocyanin components. Thus, we concluded that the highly expressed genes in samples of Zhejiang province did not necessarily affect the contents of anthocyanins. Except for structural genes, the types of anthocyanins presented in plants were determined by other enzymes such as UGTs and serine carboxypeptidase genes. Thus, we speculated that the highly expressed and correlative genes of 2 UGTs and 2 serine carboxypeptidases also play key roles in anthocyanin biosynthesis in *D. officinale*.

Here, we proposed a sinapoyl anthocyanin biosynthesis pathway in *D. officinale*, which was described below. First, cyanidin and delphinidin were synthesized via the structural genes of the anthocyanin pathway. Chalcone flavonone isomerase-like and hydroxycinnamoyltransferase 1 may be the key enzyme to determine the total anthocyanin content. Then, cyanidin and delphinidin were glycosylated by UGTs such as UGT-83A1 and UGT-88B1 isoform X1 to attach the glycosyl to specific positions. Third, via the activity of serine carboxypeptidase-like 18 and serine carboxypeptidase-like 18 isoform X3, sinapoylation of cyanidin and delphinidin were achieved.

## 5. Conclusions

Quantitative and qualitative analyses showed that the anthocyanins in *D. officinale* plants from different places were different. The total anthocyanin concentration in samples from Guangxi was the highest. This result was consistent with the apparent characteristics of this herbal plant. By transcriptomic analysis, we revealed a possible biosynthetic pathway for the anthocyanins identified in this study; anthocyanidin synthesis-related genes, UGTs, and serine carboxypeptidase were involved in this pathway. The possible drug targets of anthocyanin were predicted by molecular docking. However, identification of the exact functions or roles of these analyzed genes required further investigation. In vitro enzyme activity assays and the exact pharmacological activity of anthocyanins needed to be verified.

## Figures and Tables

**Table 1:** The fragmentation regular of the identified anthocyanins in *D. officinale*.

Peak No.	Rt (time)	MS	MS^2^	Anthocyanin identification	Molecular formula
1	10.944	979.01	817.12, 655.05, 449.09, 287.09	Cyanidin 3-[2-(glucosyl)-6-(sinapoyl)glucoside]-5-glucoside	C_44_H_51_O_25_^+^
2	16.604	595.05	433.05, 415.05, 397.13, 295.05	Cyanidin 3-O-rutinoside	C_27_H_31_O_15_^+^
3	19.285	1202.04	832.96, 671.07, 517.05, 465.04, 303.18	Delphinidin 3-glucoside-7, 3′-di-[6-(sinapoyl)glucoside]	C_55_H_61_O_30_^+^
4	20.380	817.05	655.11, 449.11, 287.09, 245.16	Cyanidin 3-[6-(sinapoyl)glucoside]-5-glucoside	C_38_H_41_O_20_^+^
5	22.483	1185.01	1022.94, 817.09, 654.91, 449.09, 287.06	Cyanidin 3-[6-sinapoyl-2-O-(2-(sinapoyl)glucosyl)-glucoside]-5-glucoside	C_55_H_61_O_29_^+^
6	29.950	655.05	449.03, 287.11	Cyanidin 3-[6-(sinapoyl)glucoside]	C_32_H_31_O_15_^+^
7	30.945	1022.8	655.14, 449.27, 278.86	Cyanidin 3-[6-sinapoyl-2-O-(2-(sinapoyl)glucosyl)-glucoside]	C_49_H_51_O_24_^+^

**Table 2:** Results of molecular docking information of protein targets and active component performed by Schrödinger's maestro software.

Compound	Target	PDB ID	Docking score	Glide gscore	Glide emodel	RMSD
Cyanidin 3-[2-(glucosyl)-6-(sinapoyl)glucoside]-5-glucoside	CTSD	4OD9	-7.562	-7.562	-100.598	1.8419
	CA9	6FE0	-6.530	-6.530	-70.828	1.0379

## Data Availability

LC-MS datas and the anthocyanidin related targets were included in supplemental data.

## Supplementary Materials

Supplementary Materials Figures S1-7: the fragmentation regular of 7 identified anthocyanins. Table S1: the common targets of the identified anthocyanins and breast cancer in *D. officinale.* Table S2a: the Pearson correlation coefficient of the related genes and metabolites. Table S2b: list of abbreviations.
