# Supplementary material for: Network Analysis of Transcriptome and LC-MS Reveals a Possible Biosynthesis Pathway of Anthocyanins in Dendrobium officinale
Source: Biomed Res Int. 2020 Apr 29;2020:6512895. doi: 10.1155/2020/6512895 (PMC7210514; doi:10.1155/2020/6512895)
Supplement: Supplementary Materials — Figures S1-7: the fragmentation regular of 7 identified anthocyanins. Table S1: the common targets of the identified anthocyanins and breast cancer in D. officinale. Table S2a: the Pearson correlation coefficient of the related genes and metabolites. Table S2b: list of abbreviations. [file 6512895.f1.docx]

**Supplementary Materials**


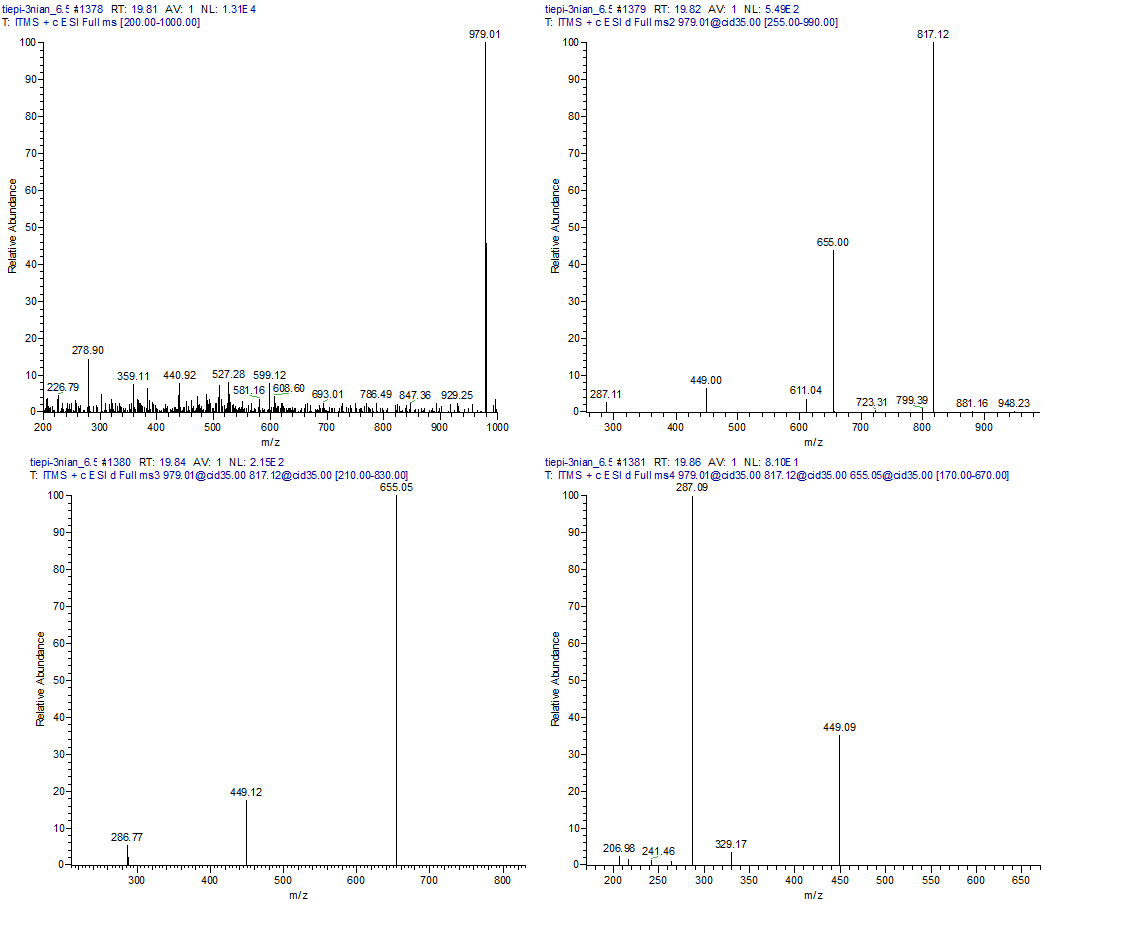


Figure S1 The fragmentation regular of cyanidin 3-[2-(glucosyl)-6-(sinapoyl)glucoside]-5-glucoside.


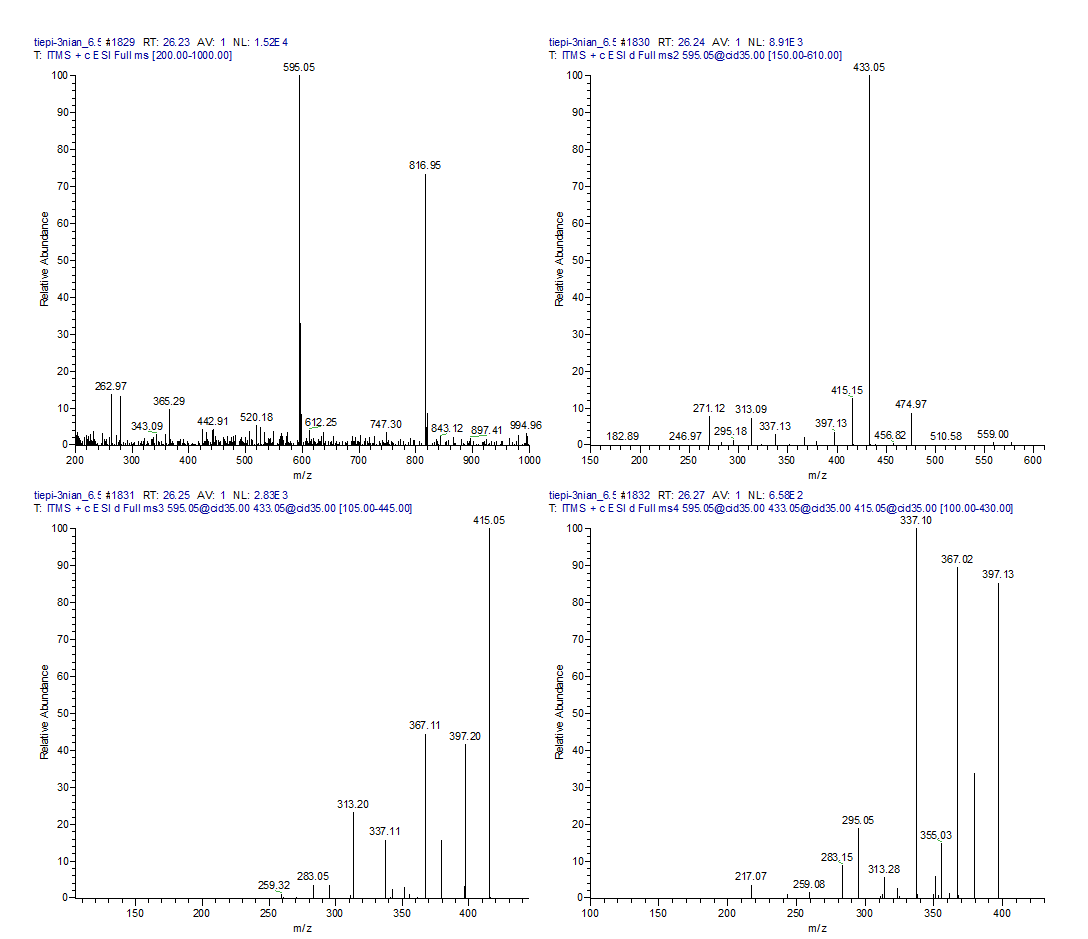


Figure S2 The fragmentation regular of cyanidin 3-O-rutinoside.


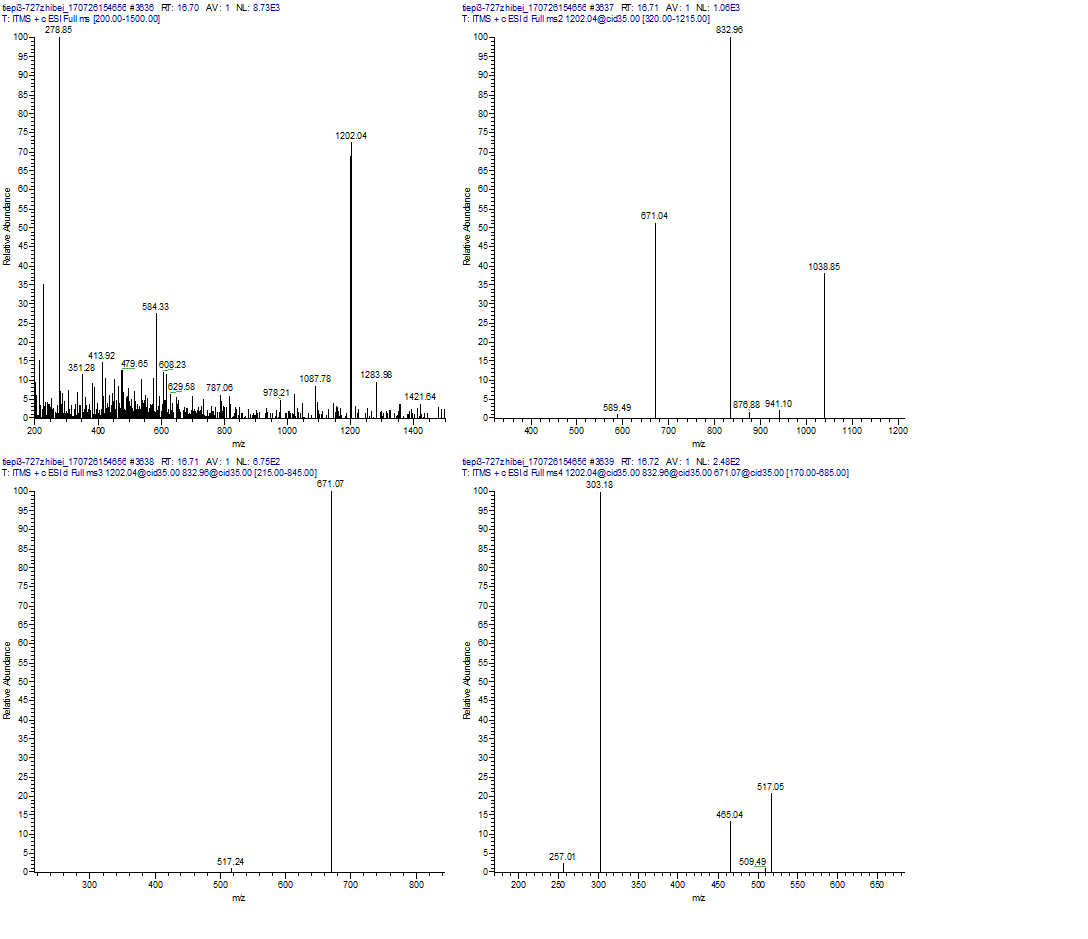


Figure S3 The fragmentation regular of delphinidin 3-glucoside-7, 3'-di-[6-(sinapoyl)glucoside].


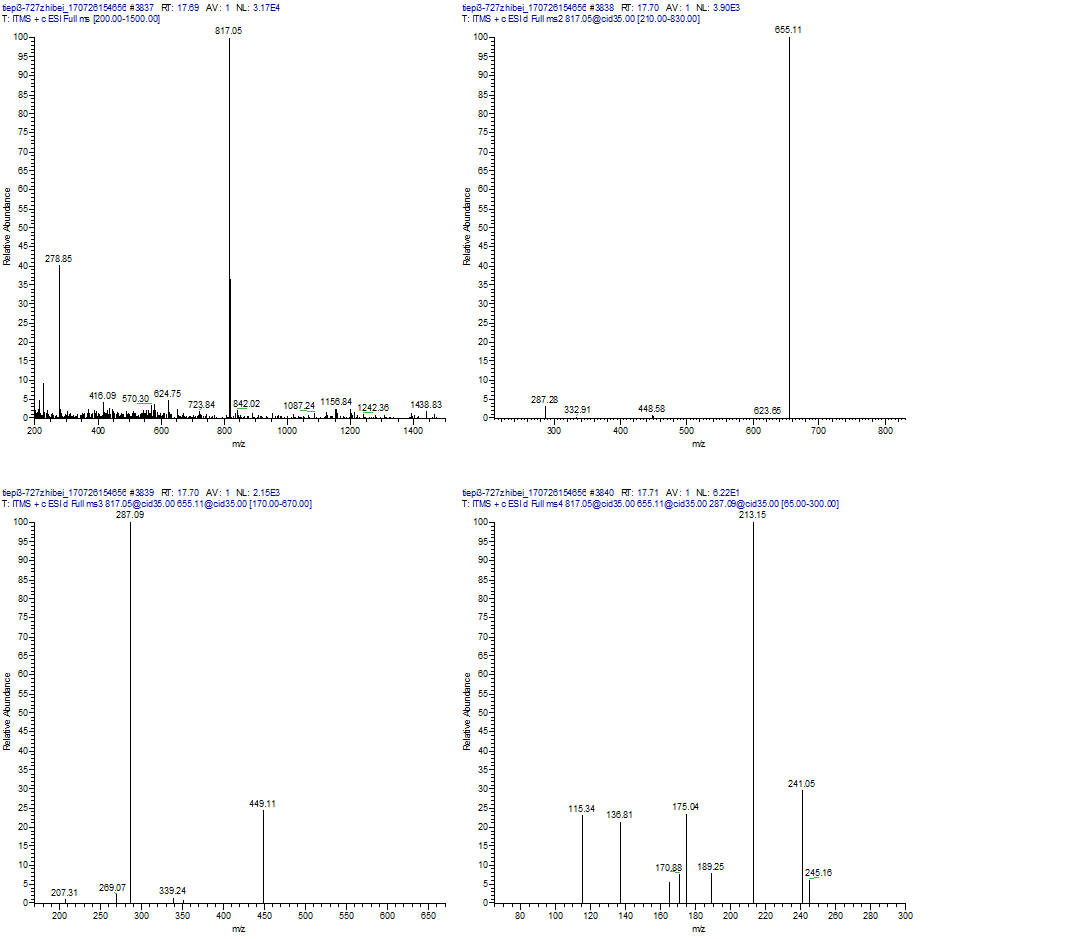


Figure S4 The fragmentation regular of cyanidin 3-[6-(sinapoyl)glucoside]-5-glucoside.


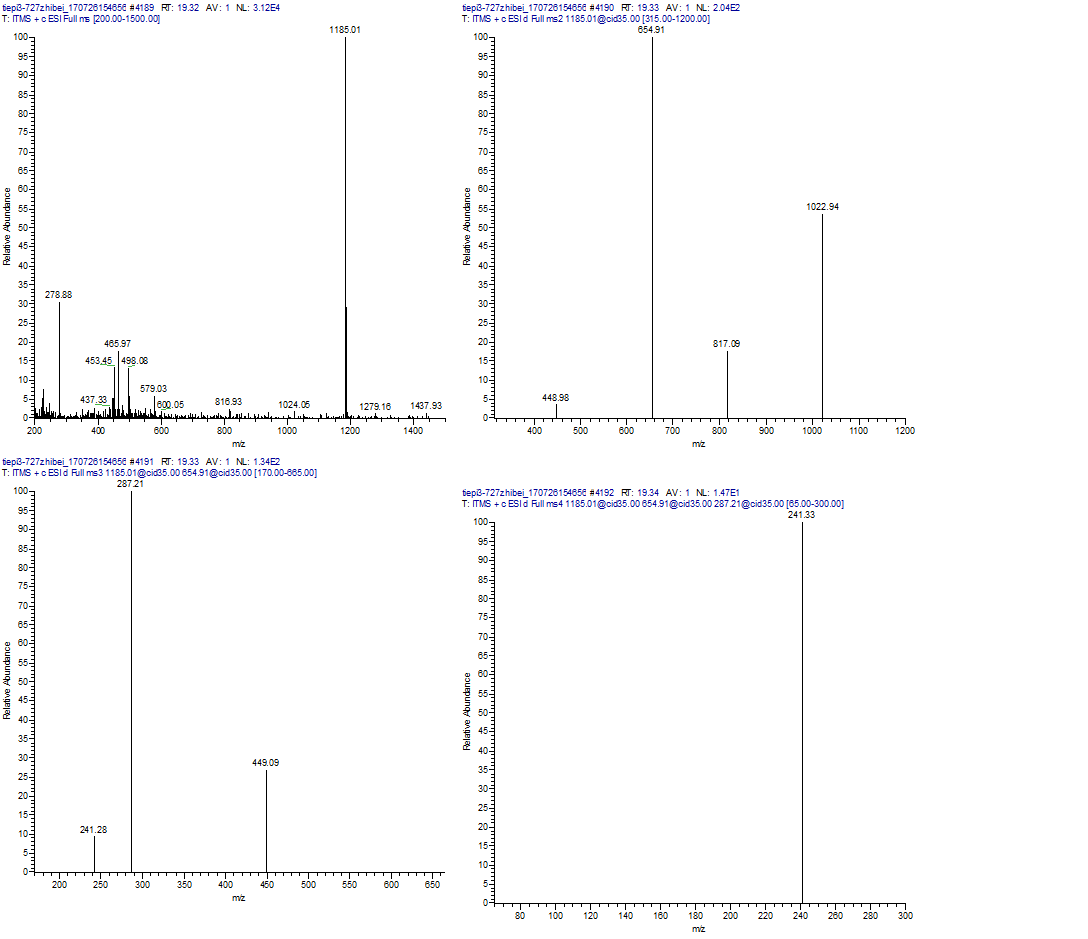


Figure S5 The fragmentation regular of cyanidin 3-[6-sinapoyl-2-O-(2-(sinapoyl)glucosyl)-glucoside]-5-glucoside.


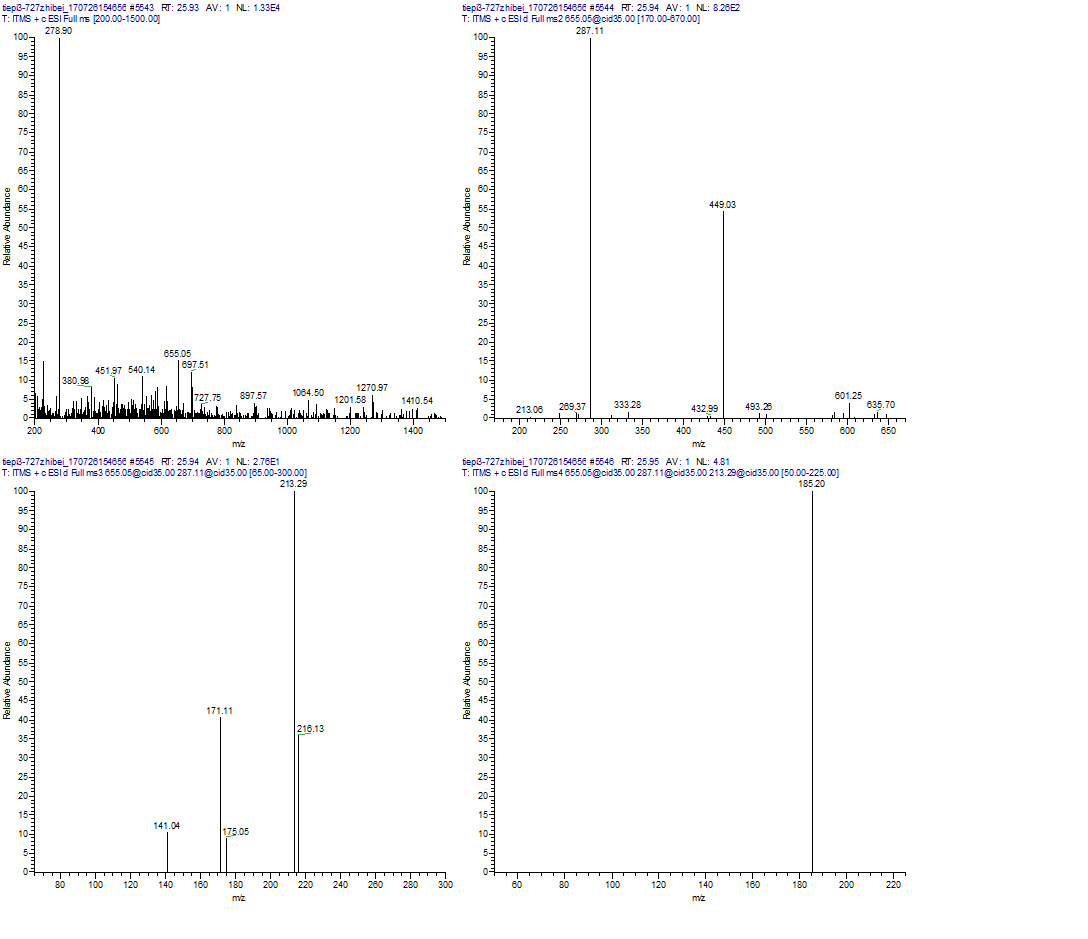


Figure S6 The fragmentation regular of cyanidin 3-[6-(sinapoyl)glucoside].


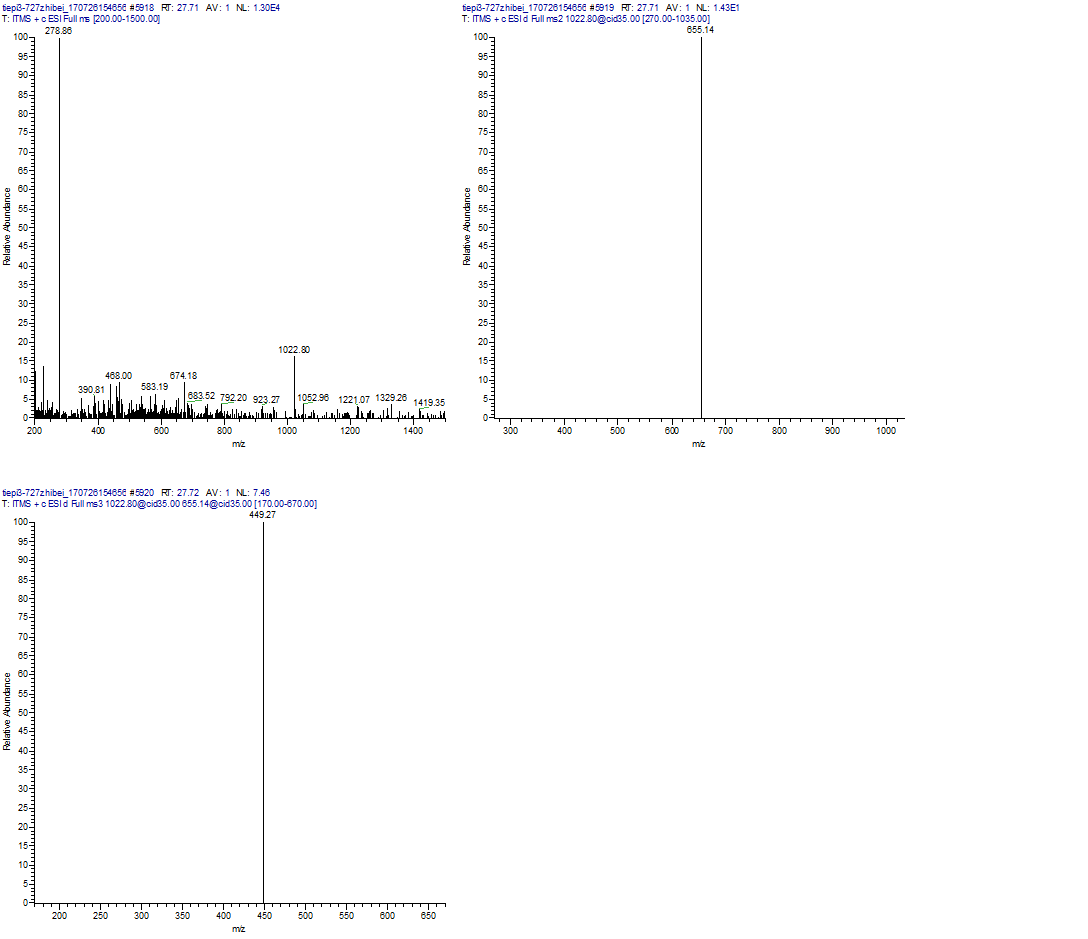


Figure S7 The fragmentation regular of cyanidin 3-[6-sinapoyl-2-O-(2-(sinapoyl)glucosyl)-glucoside].

Table S1 The common targets of the identified anthocyanins and breast cancer in *D. officinale*.

| Peak No. | Anthocyanidin Identification | targets |
| --- | --- | --- |
| 1 | Cyanidin 3-[2-(glucosyl)-6-(sinapoyl)glucoside]-5-glucoside | CA9, CTSD, FGF1, FGF2, TYMS, VEGFA |
| 2 | Cyanidin 3-O-rutinoside | CA9, MMP1, MMP13, TNF, TOP1 |
| 3 | Delphinidin 3-glucoside-7, 3'-di-[6-(sinapoyl)glucoside] | ABCB1, ABCC1, ABCG2, FGF1, FGF2, LGALS3, PRKCA, PTGS2, VEGFA |
| 4 | Cyanidin 3-[6-(sinapoyl)glucoside]-5-glucoside | CA9, CTSD, HRAS, JUN, LGALS3, PARP1, TNF, TOP1 |
| 5 | Cyanidin 3-[6-sinapoyl-2-O-(2-(sinapoyl)glucosyl)-glucoside]-5-glucoside | CA9, CTSD |
| 6 | Cyanidin 3-[6-(sinapoyl)glucoside] | TOP1 |
| 7 | Cyanidin 3-[6-sinapoyl-2-O-(2-(sinapoyl)glucosyl)-glucoside] | EGFR, ERBB2 |

Table S2a The Pearson correlation coefficient of the related genes and metabolites.

| Genes | Metabolites | Pearson correlation coefficient | Genes | Metabolites | Pearson correlation coefficient |
| --- | --- | --- | --- | --- | --- |
| CYP98A2 | Peak5 | 0.954** | SC50X2 | Peak5 | 0.77* |
| 89B1like | Peak6 | 0.944** | SC36 | Peak1 | 0.761* |
| CYP98A2 | Peak4 | 0.926** | HCT1 | Peak3 | 0.751* |
| SC18X3 | Peak7 | 0.907** | LAD | Peak4 | 0.749* |
| SC5 | Peak3 | 0.877** | 83A1 | Peak6 | 0.748* |
| HCT1 | Peak1 | 0.86** | CHI | Peak6 | 0.745* |
| SC42 | Peak3 | 0.854** | SC27 | Peak5 | 0.741* |
| SC42 | Peak7 | 0.846** | SC33X2 | Peak5 | 0.735* |
| SC5 | Peak7 | 0.843** | SC2 | Peak5 | 0.734* |
| SC5 | Peak1 | 0.841** | 83A1 | Peak1 | 0.734* |
| SC18X3 | Peak1 | 0.838** | 83A1 | Peak3 | 0.72* |
| SC42X5 | Peak3 | 0.83** | HCT1 | Peak2 | 0.719* |
| 89B1like | Peak2 | 0.807** | SC18X3 | Peak4 | 0.715* |
| SC18 | Peak2 | 0.801** | SC18 | Peak6 | 0.712* |
| SC42X5 | Peak2 | 0.801** | SC50X2 | Peak4 | 0.706* |
| SC42 | Peak1 | 0.796* | LAD | Peak5 | 0.704* |
| SC2 | Peak4 | 0.793* | SC42X5 | Peak7 | 0.699* |
| HCT1 | Peak7 | 0.792* | 88B1X1 | Peak3 | 0.69* |
| SC33X2 | Peak4 | 0.788* | SC34 | Peak2 | 0.685* |
| SC42 | Peak2 | 0.788* | 89B1like | Peak5 | 0.683* |
| SC5 | Peak2 | 0.786* | SC27 | Peak6 | 0.678* |
| SC18X3 | Peak3 | 0.784* | 73C3 | Peak3 | 0.674* |
| SC18 | Peak3 | 0.778* | SC35 | Peak4 | 0.673* |
| SC18 | Peak1 | 0.777* | SC34 | Peak6 | 0.668* |
| 83A1 | Peak2 | 0.771* | 73C3 | Peak2 | 0.668* |

**p*-value < 0.05

***p*-value < 0.01

Table S2b List of abbreviations.

| Abbreviation | Full name |
| --- | --- |
| DFR | dihydroflavonol 4-reductase-like |
| CYP75B1X2 | flavonoid 3'-monooxygenase-like isoform X2 |
| FLS | flavonol synthase/flavanone 3-hydroxylase-like |
| HCT1 | hydroxycinnamoyltransferase 1 |
| LAD | leucoanthocyanidin dioxygenase-like |
| NO3D | naringenin%2C2-oxoglutarate 3-dioxygenase-like |
| pCHI | probable chalcone--flavonone isomerase 3 isoform X1 |
| CYP73A | trans-cinnamate 4-monooxygenase |
| CYP73AL | trans-cinnamate 4-monooxygenase-like |
| CHI | chalcone--flavonone isomerase-like |
| HCT1L | hydroxycinnamoyltransferase 1-like |
| 5AT | anthocyanin 5-aromatic acyltransferase-like |
| BBS | bibenzyl synthase |
| CHS8 | chalcone synthase 8 |
| CYP98A2 | cytochrome P450 98A2 |
| DFRL | dihydroflavonol 4-reductase-like |
| CYP75B1X1 | flavonoid 3'-monooxygenase-like isoform X1 |
| 708A6 | UDP-glycosyltransferase 708A6-like |
| 71K2 | UDP-glycosyltransferase 71K2-like |
| 72B3 | UDP-glycosyltransferase 72B3-like |
| 73C1 | UDP-glycosyltransferase 73C1-like |
| 73C3 | UDP-glycosyltransferase 73C3-like |
| 73D1 | UDP-glycosyltransferase 73D1-like |
| 83A1 | UDP-glycosyltransferase 83A1-like |
| 86A1 | UDP-glycosyltransferase 86A1 |
| 88B1X1 | UDP-glycosyltransferase 88B1 isoform X1 |
| 89B1 | UDP-glycosyltransferase 89B1 |
| 89B1like | UDP-glycosyltransferase 89B1-like |
| SC18 | serine carboxypeptidase-like 18 |
| SC18X3 | serine carboxypeptidase-like 18 isoform X3 |
| SC2 | serine carboxypeptidase-like 2 |
| SC27 | serine carboxypeptidase-like 27 |
| SC33X2 | serine carboxypeptidase-like 33 isoform X2 |
| SC34 | serine carboxypeptidase-like 34 |
| SC35 | serine carboxypeptidase-like 35 |
| SC36 | serine carboxypeptidase-like 36 |
| SC3X4 | serine carboxypeptidase-like 3 isoform X4 |
| SC42 | serine carboxypeptidase-like 42 |
| SC42X5 | serine carboxypeptidase-like 42 isoform X5 |
| SC5 | serine carboxypeptidase-like 5 |
| SC50X2 | serine carboxypeptidase-like 50 isoform X2 |
| SC51 | serine carboxypeptidase-like 51 |
| SC7 | serine carboxypeptidase-like 7 |
